# Effectiveness of Desensitizing Dentifrices and 980 nm Diode Laser in Occluding Dentinal Tubules: An In Vitro Scanning Electron Microscopy Study

**DOI:** 10.7759/cureus.90191

**Published:** 2025-08-15

**Authors:** Akansha Pushkar, Panna Mangat, Ieeshan Farooq Shah, Subiya Haque, Aisha S Baig, Duddu Sravanteja, Seema Gupta

**Affiliations:** 1 Department of Conservative Dentistry and Endodontics, Kalka Dental College, Meerut, IND; 2 Department of Orthodontics, Kothiwal Dental College and Research Centre, Moradabad, IND

**Keywords:** dentine hypersensitivity, desensitizing agents, diode laser, scanning electron microscopy, tubule

## Abstract

Introduction: This in vitro study aimed to compare the efficacy of three desensitizing dentifrices, alongside a 980 nm diode laser, in occluding dentinal tubules to reduce dentine permeability and alleviate hypersensitivity. The primary objective was to assess tubule occlusion using scanning electron microscopy (SEM). The secondary objectives were to evaluate the relative effectiveness of each treatment in reducing dentine permeability and to compare their potential to mitigate hypersensitivity symptoms by occluding the dentinal tubules.

Materials and methods: This in vitro study was conducted on 50 extracted human first premolar teeth that were sectioned into 2 mm dentinal discs. The discs were ultrasonicated to remove the smear layer and randomly allocated into five groups (n = 10): Group 1 (control, brushed with distilled water; Merck Life Science Pvt. Ltd., Mumbai, India), Group 2 (brushed with Emoform, Dr. Wild & Co. AG, Muttenz, Switzerland), Group 3 (brushed with Propolis, Apis India Ltd., New Delhi, India), Group 4 (brushed with Sensodyne Repair and Protect, GlaxoSmithKline Consumer Healthcare, Brentford, UK), and Group 5 (treated with a 980 nm diode laser, Biolase Epic X, Biolase Inc., Irvine, USA). Groups 1-4 were brushed twice daily for seven days with a soft-bristled toothbrush (Colgate SlimSoft, Colgate-Palmolive India Ltd., Mumbai, India) using the respective slurries. Group 5 was lased at 0.8 W in continuous wave mode for 10 s. Specimens were gold-palladium-coated and analyzed by SEM (ZEISS EVO 18, Carl Zeiss AG, Oberkochen, Germany) at 5000x magnification to calculate the percentage of occluded tubules.

Results: The Kruskal-Wallis test showed significant differences in tubule occlusion (p = 0.001). The laser group demonstrated the highest occlusion rate, followed by the Sensodyne Repair and Protect, propolis-based dentifrice, and Emoform groups, all of which significantly outperformed the control group (Dunn’s test, p < 0.05). The laser group exhibited superior consistency, while Sensodyne Repair and Protect showed robust occlusion compared with the other dentifrices.

Conclusion: The 980 nm diode laser was the most effective in occluding dentinal tubules, followed by Sensodyne Repair and Protect, propolis-based dentifrice, and Emoform. These treatments offer potential for managing dentine hypersensitivity, with the laser providing rapid occlusion and Sensodyne Repair and Protect as a practical alternative. Further in vivo studies are required to validate its clinical efficacy.

## Introduction

Dentine hypersensitivity (DH) is a prevalent clinical condition characterized by sharp, transient pain arising from exposed dentin in response to various stimuli, such as thermal, chemical, tactile, or osmotic triggers [1]. This condition significantly impacts patients' quality of life, often interfering with daily activities, such as eating, drinking, and oral hygiene practices. Pain is typically localized to the facial surfaces of teeth, particularly near the cervical region, and is most commonly observed in premolars and canines [1,2]. The etiology of DH is multifactorial, with factors such as enamel erosion, gingival recession, and abrasive oral hygiene practices contributing to dentin exposure [2]. In recent years, the rise in erosive lifestyles, particularly among younger adults, has led to an increase in tooth wear and subsequent dentine hypersensitivity [2,3].

The hydrodynamic theory is the most widely accepted explanation for DH-related pain [4]. This theory posits that external stimuli induce fluid movement within dentinal tubules, activating nociceptors at the pulp-dentine interface and resulting in characteristic sharp pain [4]. Myelinated A-beta and some A-delta nerve fibers are primarily responsible for transmitting this pain response [2]. Effective management of DH aims to provide immediate and sustained relief, ideally through non-invasive, non-irritating, and aesthetically acceptable methods [3].

Two primary approaches exist for treating DH: interference with nerve transmission and occlusion of the dentinal tubules. The first approach often involves potassium-based desensitizing agents, such as potassium nitrate, which depolarize nerve fibers to reduce pain transmission [5]. However, this method offers only temporary relief, as potassium concentrations diminish without continuous application [5]. Desensitizing dentifrices containing potassium salts works by reducing intradental nerve excitability rather than physically blocking tubules, often requiring weeks to achieve noticeable effects [5].

The second approach focuses on occluding dentinal tubules to prevent fluid movement. Agents such as calcium sodium phosphosilicate (NovaMin) and strontium or stannous salts are commonly used in dentifrices to achieve this [6]. For instance, novamin reacts with saliva to form a hydroxycarbonate apatite layer, effectively sealing the exposed tubules [6]. However, strontium salts are less suitable for daily use, and stannous salts may cause tooth staining and poor taste [7]. Propolis, a natural resinous substance produced by bees, has also shown promise in reducing DH by forming crystals that adhere to dentine surfaces and occlude tubules, leveraging its bioflavonoid content and adhesive properties, similar to dental resins [8].

Laser therapy is an advanced treatment modality for DH, acting by either reducing pulpal nerve excitability or occluding tubules through protein coagulation and surface melting [9]. Diode lasers, particularly at 810 nm and 980 nm, have demonstrated the ability to partially or fully seal dentinal tubules, offering a potentially longer-lasting solution, albeit at a higher cost than desensitizing agents [10].

This in vitro study aimed to compare the efficacy of three desensitizing agents, alongside a 980 nm diode laser, in occluding dentinal tubules, using scanning electron microscopy (SEM) to evaluate their effectiveness in reducing dentin permeability and alleviating hypersensitivity. This study aimed to assess their effectiveness in reducing dentine hypersensitivity by examining their ability to occlude dentinal tubules, thereby alleviating pain triggered by external stimuli, evaluated using SEM and other appropriate analytical techniques. The secondary objectives of this study were to evaluate the effectiveness of each desensitizing agent and the 980 nm diode laser in reducing dentin permeability and achieving tubule occlusion, utilizing SEM and other relevant analytical methods. Additionally, the study compared the relative efficacy of these desensitizing agents and laser therapy in reducing dentine permeability, providing insights into their potential to prevent fluid movement within the dentinal tubules and mitigate hypersensitivity symptoms effectively.

## Materials and methods

Study design and setting

This in vitro study employed a randomized, controlled experimental design. The study was conducted over six months, from January 2024 to March 2025, in the Department of Conservative Dentistry and Endodontics at Kalka Dental College, Meerut, Uttar Pradesh, India. The SEM analyses were performed at the Department of Research and Innovation, Uttaranchal University, Dehradun, Uttarakhand, India. The study protocol adhered to the ethical standards of the 1964 Declaration of Helsinki, as revised in 2008, and was approved by the Institutional Ethical Committee of the Kalka Dental College, Meerut (Approval No. KDC/LTR/2023/0109). As an in vitro study using extracted human premolar teeth, informed consent was not required; however, the teeth were sourced from the Department of Oral and Maxillofacial Surgery with departmental approval, ensuring compliance with institutional guidelines.

Sample size estimation

The sample size was calculated using G*Power software (version 3.1.9.6, programmed by Franz Faul, University of Kiel, Kiel, Germany). With an estimated effect size of 0.55, based on prior research comparing occluded dentinal tubules between desensitizing agents and laser applications, assuming an alpha error of 0.5% and 80% statistical power, a minimum of 40 samples (10 per group) was required [11].

Eligibility

Eligible samples included extracted human premolar teeth with intact root surfaces that were free from caries, restorations, or structural defects. The exclusion criteria were teeth with root caries, wasting diseases, restorations, hypoplasia, bleaching, fluorosis, fractures, and eroded or cracked teeth. The discontinuation criteria included specimens with improper thickness (<2 mm), damage during disc preparation, or sample loss, which was addressed by replacing the affected samples with fresh specimens to maintain the required sample size.

Methodology

Fifty human first premolar teeth extracted for orthodontic purposes were collected from the Department of Oral and Maxillofacial Surgery, Kalka Dental College, Meerut. The teeth were cleaned with normal saline (sodium chloride 0.9%, Parenteral Drugs India Ltd., Indore, India) to remove blood and saliva, washed with distilled water, and stored in 10% formalin (formaldehyde solution 10%; Merck Life Science Pvt. Ltd., Mumbai, India) at room temperature. Each tooth was scaled using an ultrasonic scaler (Woodpecker UDS-J, Guilin Woodpecker Medical Instrument Co., Ltd., Guilin, China) and sectioned mesiodistally with a double-sided diamond disk (DFS Diamond disc, DFS-Diamon GmbH, Riedenburg, Germany) to obtain a 2 mm thick dentinal disc from the mid-coronal dentine, and verified using a digital Vernier caliper (Mitutoyo Absolute Digimatic Caliper, Mitutoyo Corporation, Kawasaki, Japan). Discs were ultrasonicated in distilled water for 12 min using an ultrasonic cleaner (Labman LMUC-4, Labman Scientific Instruments Pvt. Ltd., Chennai, India) to remove the smear layer and open the dentinal tubules.

Fifty specimens were randomly allocated into five groups (n = 10 each): Group 1 (control, brushed with distilled water; Merck Life Science Pvt. Ltd.), Group 2 (brushed with Emoform; Dr. Wild & Co. AG, Muttenz, Switzerland), Group 3 (brushed with Propolis, Apis India Ltd., New Delhi, India), Group 4 (brushed with Sensodyne Repair and Protect, GlaxoSmithKline Consumer Healthcare, Brentford, UK), and Group 5 (lased with a 980 nm diode laser, Biolase Epic X, Biolase Inc., Irvine, CA, USA).

Groups 1-4 were brushed twice daily for seven days with their respective treatments (dentifrice slurries prepared by diluting 2 g of dentifrice in 6 mL of distilled water for Groups 2-4). Each dentinal specimen was brushed using a soft-bristled manual toothbrush (Colgate SlimSoft, Colgate-Palmolive India Ltd., Mumbai, India) to minimize mechanical abrasion of the dentine surface. Brushing was performed by a single trained operator to ensure uniformity in the pressure and technique. For each brushing session, approximately 0.5 mL of the prepared slurry (or distilled water for Group 1) was applied to the toothbrush bristles. The specimen was brushed with linear back-and-forth strokes at a standardized force of approximately 200 g and monitored using a digital scale during training to calibrate the operator’s hand pressure. Each specimen was brushed for two minutes per session, twice daily (morning and evening, with a 12-hour interval), for a total of seven days, simulating a typical clinical desensitizing regimen. After each brushing session, the specimens were rinsed gently with 5 mL of distilled water to remove residual slurry or debris, and stored in distilled water at room temperature until the next session. For the control group (Group 1), the specimens were brushed with distilled water alone, following the same brushing protocol (two minutes, twice daily for seven days), to serve as a baseline for comparison without active desensitizing agents.

Group 5 was treated with a 980 nm diode laser, where the laser was set to a wavelength of 980 nm, power output of 0.8 W, and continuous wave (CW) mode, with a duration of 10 s per specimen using a 200 µm fiber optic tip. The laser tip was held perpendicular to the dentinal surface at a distance of approximately 1 mm in non-contact mode, applied in a sweeping motion to uniformly cover the 2 mm dentinal disc surface (~3.14 mm²), yielding an energy density of approximately 25.5 J/cm². Safety measures included operator protective eyewear, pre-session laser calibration, and a controlled environment to minimize unintended exposure risks. These settings, based on prior evidence [10], were selected for their efficacy in sealing dentinal tubules by coagulating proteins in the dentinal fluid and forming an amorphous sealed layer, thus reducing permeability.

Outcome assessment

**Figure 1:**
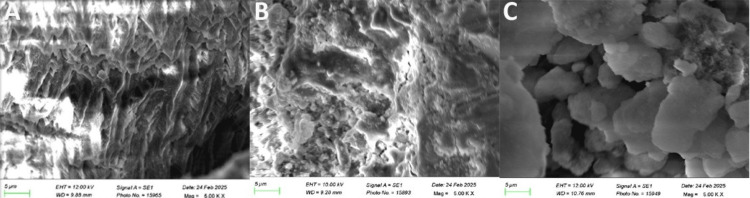
Scanning electron microscope images of dentinal samples at 5000x. A). Group 1 (control, brushed with distilled water), B). Group 2 (brushed with Emoform), C). Group 3 (brushed with Propolis). These images represent samples from the study.

**Figure 2:**
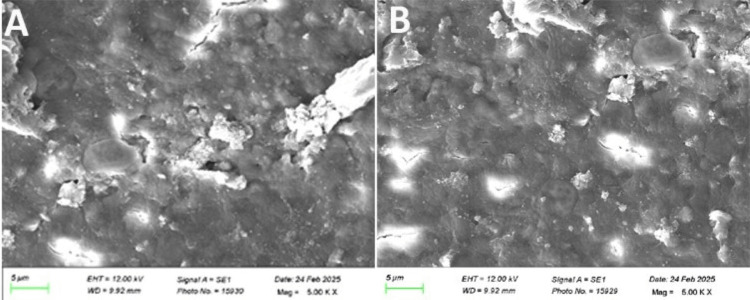
Scanning electron microscope images of dentinal samples at 5000x. A). Group 4 (brushed with Sensodyne Repair and Protect), B). Group 5 (lased with a 980 nm diode laser). These images represent samples from the study.

Treated specimens were mounted on aluminum stubs and gold-palladium coated using a sputter coater (Quorum Q150R ES, Quorum Technologies Ltd., Lewes, UK) and analyzed using a SEM (ZEISS EVO 18, Carl Zeiss AG, Oberkochen, Germany) at 5000x magnification (Figure 1, Figure 2).

The percentage of occluded tubules was calculated as the number of occluded tubules divided by the total number of tubules multiplied by 100.

Calibration and reliability assessment

To ensure consistency, a single operator performed all specimen preparations and treatments and was trained by a senior faculty member from the Department of Conservative Dentistry. SEM analysis was conducted by a blinded examiner to minimize bias. Inter-examiner reliability was assessed using Cohen’s kappa coefficient, which yielded a value of 0.85, indicating excellent agreement. The Vernier caliper and ultrasonic cleaner were calibrated according to the manufacturer’s guidelines before use.

Statistical analysis

Data were entered into Microsoft Excel 2007 (Microsoft Corporation, Redmond, WA, USA) and analyzed using SPSS Statistics version 23.0 (IBM Corp., Armonk, NY, USA). Descriptive statistics, including mean and standard deviation, were calculated for the percentage of occluded tubules. The normality of the data was assessed using the Shapiro-Wilk test, and non-normal data distribution for the blocked percentage of dentinal tubules was confirmed by a histogram/Q-Q plot. Intergroup comparisons of the total dentinal tubules were performed using one-way analysis of variance (ANOVA), and the Kruskal-Wallis test was used to determine the percentage of blocked dentinal tubules. Post-hoc analysis with Dunn’s test was used for significant intergroup comparisons. Statistical significance was set at p < 0.05.

## Results

The one-way ANOVA test comparing the total dentinal tubule counts across the five experimental groups showed no statistically significant differences (p = 0.071). While numerical trends were observed, with Group 5 demonstrating the highest mean dentinal tubule counts (83.5 ± 8.03) and Group 1 the lowest (69.4 ± 8.75), however, the difference was not statistically significant (p > 0.05). All groups showed comparable ranges, suggesting similar variability in baseline dentinal tubule characteristics. These results implied that the baseline conditions did not significantly influence the total number of dentinal tubules present (Table 1).

**Table 1:** Comparison of total number of dentinal tubules between groups by one-way analysis of variance (ANOVA) test. p-value > 0.05: non-significant. Data is presented in form of mean ± standard deviation (SD).

Groups	N	Minimum	Maximum	Range	Mean ± SD	F value	p-value
Group 1 (Control)	10	56	86	30	69.40 ± 8.75	2.58	0.071
Group 2 (Emoform)	10	50	82	32	70.10 ± 10.31		
Group 3 (Propolis)	10	56	88	32	72.40 ± 9.65		
Group 4 (Sensodyne Repair and Protect)	10	64	90	26	77.40 ± 7.43		
Group 5 (980 nm Diode laser)	10	70	95	25	83.50 ± 8.03		

The Kruskal-Wallis test revealed highly significant differences in dentinal tubule occlusion among the groups (p = 0.001). Group 1 showed a markedly lower blockage than the experimental groups. Notably, Group 5 demonstrated the highest occlusion rate (88.66 ± 6.14%), suggesting both superior efficacy and consistency. This was followed by Groups 4, 3, and 2. These findings demonstrate that all experimental treatments significantly outperformed the control, with Group 5 emerging as the most effective intervention for dentinal tubule occlusion (Table 2).

**Table 2:** Post-application comparison of percentage of blocked dentinal tubules between groups by Kruskal-Wallis test. *p-value < 0.05: significant. Data is presented in form of mean ± standard deviation (SD).

Groups	N	Median	Mean Rank	Range	Mean ± SD	Chi value	p-value
Group 1 (Control)	10	22.75	5.50	16.00	22.72 ± 6.17	31.19	0.001*
Group 2 (Emoform)	10	72.05	20.35	38.00	70.34 ± 15.48		
Group 3 (Propolis)	10	78.90	26.05	28.00	81.50 ± 12.16		
Group 4 (Sensodyne Repair and Protect)	10	84.95	30.10	26.00	84.01 ± 6.81		
Group 5 (Diode laser)	10	87.00	38.50	17.70	88.66 ± 6.14		

Post-hoc Dunn's test revealed significant differences in treatment efficacy between the groups. All experimental groups showed significantly higher mean differences than Group 1 (control; p < 0.05), with Group 5 demonstrating the greatest effect (-65.94, p = 0.001). While Groups 2, 3, and 4 did not differ significantly (p > 0.05), Group 5 showed superior performance compared to Group 2 (p = 0.008). These findings suggest that, while all experimental interventions outperformed the control, Group 5 might offer the most effective solution, warranting further investigation into its optimal application parameters. The non-significant differences among Groups 2, 3, and 4 suggested comparable efficacy among these treatments (Table 3).

**Table 3:** Pairwise comparison using post-hoc analysis with Dunn test. *p-value < 0.05: significant, Group 1: control, brushed with distilled water, Group 2: brushed with Emoform, Group 3: brushed with Propolis, Group 4: brushed with Sensodyne Repair and Protect, and Group 5: lased with a 980 nm diode laser.

Pairwise group	Mean difference	Standard Error	Test Statistic	p-value
Group 1 - Group 2	-47.62	6.52	-2.42	0.016*
Group 1 - Group 3	-58.78	6.52	-3.56	0.001*
Group 1 - Group 4	-61.29	6.52	-4.30	0.001*
Group 1 - Group 5	-65.94	6.52	-5.06	0.001*
Group 2 - Group 3	-11.16	6.52	-1.14	0.253
Group 2 - Group 4	-13.67	6.52	-1.89	0.059
Group 2 - Group 5	-18.32	6.52	-2.65	0.008*
Group 3 - Group 4	-2.51	6.52	-0.74	0.457
Group 3 - Group 5	-7.16	6.52	-1.50	0.133
Group 4 - Group 5	-4.65	6.52	-0.76	0.447

## Discussion

The results of the study demonstrated significant differences in tubule occlusion among the groups, with the 980 nm diode laser achieving the highest occlusion rate (88.66 ± 6.14%), followed by Sensodyne Repair and Protect, propolis-based dentifrice, and Emoform, all of which significantly outperformed the control group. These findings align with the hydrodynamic theory of dentine hypersensitivity, which suggests that occluding dentinal tubules reduces fluid movement, thereby mitigating pain triggered by external stimuli [4]. The choice of SEM was predicated on its ability to provide high-resolution, three-dimensional, non-destructive, and objective imaging. This methodology has been routinely employed in previous investigations to assess the occlusion of dentinal tubules [12].

The Emoform, containing potassium nitrate and fluoride, exhibited a moderate tubule occlusion rate, which was significantly higher than that of the control but lower than that of the laser group. Potassium nitrate reduces nerve excitability by increasing extracellular potassium ion concentration and depolarizing sensory nerve endings, whereas fluoride promotes remineralization, forming fluorapatite or calcium fluoride-like deposits that partially occlude tubules [5,7]. James et al. [13] concluded that brushing twice with potassium nitrate per day for two minutes was effective in occluding the dentinal tubules. Salian et al. [14] reported that 5% potassium nitrate was less effective than 5% NovaMin in occluding dentinal tubules.

Propolis, a natural resinous substance, showed occlusion comparable to that of Sensodyne Repair and Protect, but was less effective than the laser. Its mechanism involves the deposition of organic compounds such as flavonoids and phenolic acids, which adhere to dentine surfaces, forming a physical barrier over the tubules [8]. In addition, propolis has anti-inflammatory properties that may indirectly reduce hypersensitivity. Similar results have been reported by Naghsh et al. [8]. In contrast, Purra et al. [15] reported better efficacy of propolis than 5% potassium nitrate for DH. Propolis is a significant reservoir of chemical compounds, notably bioactive flavonoids. Research has demonstrated that flavonoids possess tissue regeneration capabilities. Furthermore, propolis has been shown to enhance the activity of numerous enzymes, promote cellular metabolism, improve circulation, and facilitate collagen synthesis, in addition to accelerating the healing process [16].

Sensodyne Repair and Protect, formulated with NovaMin (calcium sodium phosphosilicate), demonstrated robust tubule occlusion, surpassing Emoform and propolis-based dentifrice, but falling short of the laser group. This aligns with previous studies [6,17,18]. Sodium calcium phosphosilicate bioactive glass (NovaMin® technology) utilized in the Sensodyne Repair and Protect dentifrice functions as a reservoir for calcium and phosphate ions, mimicking biological apatite [17]. In dentifrice formulations, calcium, phosphate, sodium, and silica dioxide ions are incorporated into an amorphous matrix [6]. Upon exposure to an aqueous medium (such as water or saliva), sodium ions are released. This liberation results in an increase in the pH, thereby facilitating the rapid precipitation of calcium and phosphate ions, leading to the formation of a hydroxyapatite layer. Prior research has indicated that this protective layer of amorphous calcium phosphate is established within one hour of contact with a basic buffer solution [19].

The 980 nm diode laser achieved the highest occlusion rate, with post-hoc Dunn’s test confirming its superior efficacy compared to Emoform and the control. The laser seals tubules by coagulating proteins in the dentinal fluid and forming an amorphous layer, thereby reducing permeability. This mechanism is supported by Umana et al. [10], who demonstrated that 980 nm diode lasers effectively occlude tubules by melting and recrystallizing dentine surfaces at low power settings (0.8 W, as used in this study. Similar results were reported by Rodriguez et al. [20]. The authors further concluded that diode lasers of 980 nm, when used with a photoinitiator, promoted dentinal tubule obliteration with the least thermal damage. The consistency of occlusion in the laser group further underscores the precision and reliability of the laser. The lack of significant differences in total tubule counts across groups confirmed that the baseline dentine characteristics were consistent, ensuring that the observed differences in occlusion were treatment-related.

Clinical implications

The superior performance of the laser suggests that it may be the most effective intervention for rapid and consistent tubule occlusion, potentially offering immediate relief in the clinical setting. However, its high cost and requirement for specialized equipment limit its accessibility compared with dentifrices. Sensodyne Repair and Protect Deep Repair's strong performance makes it a practical alternative for home use, while propolis offers a natural option, although its long-term stability requires further investigation. The moderate efficacy of the Emoform suggests that it may be better suited for symptomatic relief rather than for robust occlusion.

Limitations and future directions

The in vitro design limited the direct extrapolation to clinical outcomes, as oral conditions (such as saliva and dietary acids) might affect treatment efficacy. Additionally, the seven-day brushing protocol might not reflect the long-term outcomes. Future studies should incorporate in vivo models and longer treatment durations to assess the durability of occlusions and clinical pain reduction. Comparative studies using other laser wavelengths (such as Nd:YAG) could further elucidate the optimal laser parameters.

## Conclusions

This in vitro study demonstrated that the 980 nm diode laser and three desensitizing agents, Emoform, propolis-based dentifrice, and Sensodyne Repair and Protect, effectively occluded dentinal tubules compared to the control; the laser showed the highest efficacy. These findings suggest that all treatments have the potential to reduce dentin permeability and alleviate hypersensitivity by limiting fluid movement within the tubules, supporting their use in managing dentine hypersensitivity. The superior performance of the laser highlights its promise for clinical applications, although cost and accessibility may favor dentifrices such as Sensodyne Repair and Protect for routine use. Further in vivo studies are needed to confirm these results under clinical conditions and evaluate their long-term efficacy.
